# Risk Factors for Reintervention in Children with Subvalvular Aortic Stenosis: A 20-Year Single-Center Study

**DOI:** 10.3390/jcdd12100413

**Published:** 2025-10-21

**Authors:** Jelena Hubrechts, Alessandra Zanfardino, Catherine Barrea, Thierry Detaille, Alain Poncelet

**Affiliations:** 1 Division of Congenital and Pediatric Cardiology, Department of Pediatrics, University Hospitals Saint-Luc, Université Catholique de Louvain, Avenue Hippocrate 10, B-1200 Brussels, Belgium; 2 Department of Cardiovascular and Thoracic Surgery, University Hospitals Saint-Luc, Université Catholique de Louvain, B-1200 Brussels, Belgiumalain.poncelet@saintluc.uclouvain.be (A.P.); 3 Department of Pediatric Intensive Care Unit, University Hospitals Saint-Luc, Université Catholique de Louvain, B-1200 Brussels, Belgium

**Keywords:** subvalvular aortic stenosis, surgical resection, risk factors of recurrence, children

## Abstract

Subvalvular aortic stenosis (SAS) is a frequent cause of left ventricular outflow tract (LVOT) obstruction in children. Surgical resection is the standard treatment, yet recurrence requiring reintervention remains a significant concern. We aim to identify risk factors associated with surgical reintervention. This retrospective study included 76 patients under 18 years who underwent surgical resection for SAS between 2000 and 2020. Preoperative, intraoperative, and postoperative data were analyzed. The mean age at initial surgery was 5.8 years. In addition to subaortic membrane resection, myomectomy was performed in 73.7% of patients. Reintervention was required in 13.1% of cases, with reintervention-free survival at 5 and 10 years of 93.4% and 89.5%, respectively. Significant predictors of reintervention included younger age and shorter stature at surgery. Myomectomy reduced postoperative gradients but was not protective against recurrence and was associated with a higher rate of conductive disorders requiring pacemakers. While surgical resection of SAS is associated with excellent survival, recurrence remains a concern, especially in younger patients and those with tunnel-like lesions. Individualized surgical planning is essential to balance recurrence risk with potential complications.

## 1. Introduction

Obstructive lesions in the left ventricular outflow tract (LVOT) can occur at different levels. After valvular aortic stenosis, subvalvular aortic stenosis (SAS) is the most prevalent condition, accounting for 20% of all fixed obstructive lesions of the LVOT [1,2]. SAS is frequently associated with other congenital heart defects, such as bicuspid aortic valve, ventricular septal defect, and Shone’s complex. SAS is a progressive disease that requires surgical treatment, although the age at intervention may vary greatly. Commonly, three different types of SAS can be distinguished: (1) membranous, representing 75–85% of cases, (2) fibromuscular ridge, and (3) diffuse fibromuscular tunnel-like lesions [1,3].

Several pathophysiological processes are believed to play a role in the onset of subaortic stenosis, including the anatomical elements that enhance turbulence and shear stress. Aortic regurgitation (AR) is a well-known complication, probably due to the thickening of the aortic valve leaflets injured by the high-velocity stream through the SAS and the restriction of the mobility of the valve secondary to the involvement of the ventricular aspect of the leaflets [4,5].

Currently, management guidelines for the pediatric population are lacking. Although surgical intervention appears to be the sole conclusive treatment, the specific criteria and optimal timing for its application remain unclear.

Recommendations range from early surgery to longer periods of observation, depending on patient characteristics [5]. Ezon et al. [6] reported that only two studies recommended surgery at diagnosis, regardless of the severity of obstruction. Brauner et al. [7] suggested that early surgery prevents AR. However, the prevention of AR alone is not a criterion for surgery. According to the 2008 American Heart Association (AHA) guidelines [8], unoperated adults with a mean gradient < 30 mmHg and without significant LV hypertrophy are recommended to be followed up annually because some of these patients will eventually require surgery. In adult patients with equivocal indications for intervention, stress testing to determine exercise capability, symptoms, electrocardiographic changes, arrhythmias, or an increase in the LVOT gradient is reasonable. Surgery seems to be indicated in symptomatic patients with peak-to-peak gradients > 50 mmHg. To the best of our knowledge, no pediatric guidelines are currently available.

Long-term complications after surgical resection include high rates of lesion recurrence and progressive aortic regurgitation. Different predictors of recurrence have been studied, including younger age at primary surgery, the presence of aortic regurgitation, higher pre- and postoperative peak gradients, short distance between the aortic valve and the subaortic membrane, anatomical variations in the outflow tract, and the presence of other congenital heart defects [2,5,7,9,10,11,12,13].

## 2. Materials and Methods

### 2.1. Aim

This single-center retrospective study aimed to identify the risk factors for surgical reintervention after successful first resection.

### 2.2. Study Population and Data Collection

This systematic retrospective chart review included all pediatric patients who had undergone primary resection or reintervention for SAS at the University Hospitals Saint-Luc Brussels between January 2000 and December 2020. We identified 76 patients aged between 0 and 18 years.

During the study period, indications for surgery were (a) peak-to-peak gradient > 50 mmHg, (b) new-onset aortic valve regurgitation in the setting of anatomical substrate or progressive aortic regurgitation and progressing peak-to-peak gradient (without the 50 mmHg threshold), and (c) the development of LV dysfunction or progressive LV dilatation (using Z-scores for body surface area).

Medical Explorer, EPIC, Intellispace, and MicroDicom were used to collect patient data.

All patient records were analyzed by an operator to extract preoperative, intraoperative, and postoperative information. Follow-up was 90% complete (8 international patients), with a median time of 66 months (IQR25-75 30–142 months).

The most recent preoperative, postoperative, and follow-up echocardiographic images and reports were reviewed. Measurements used for statistical analysis were either measurements from the patient’s echocardiographic reports or the averages of two measurements made by the same operator. Z-scores for echocardiographic parameters were based on the Boston Children’s Hospital reference values.

Peak systolic instantaneous LVOT gradient was derived from the continuous-wave Doppler LVOT peak flow velocity.

**Figure 1:**
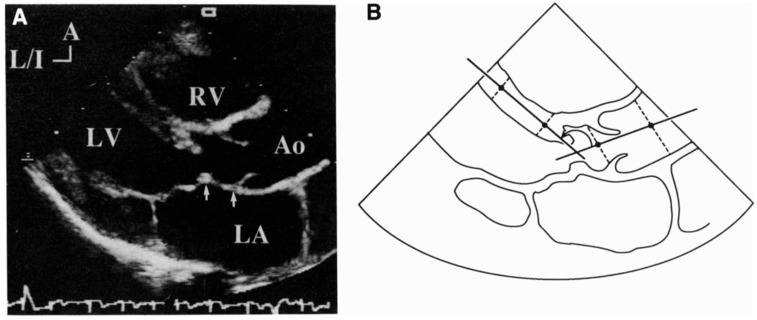
Illustration of the aortoseptal angle, adapted from Kleinert et al., 1993 [14]. (**A**) Parasternal long-axis view showing an obstructive subaortic ridge. Arrows show mitral valve–aortic valve separation. (**B**) Diagrammatic presentation of the same view demonstrating the method for measuring the angle between the long axis of the aortic root and proximal ascending aorta and the midline of the interventricular septum. A = anterior, Ao = aorta, LA = left atrium, L/I = left/inferior, LV = left ventricle, RV = right ventricle.

The aortoseptal angle was defined as the angle between a line drawn along the axis of the interventricular septum and a line drawn through the long axis of the aortic root, where a value of 180° would be a straight line from the septum to the aorta, and decreasing values would represent increasing angulation. This angle was measured in the parasternal long-axis view at end diastole (Figure 1).

Authorization was granted by the Medical Ethical Committee of Saint-Luc University Hospitals (CEHF/2022/08FEV/054).

### 2.3. Statistical Analysis

Continuous data were presented as mean ± standard deviation or median (interquartile range) for parametric and non-parametric data, respectively. The normality of the distribution was assessed using the Shapiro–Wilk test. Categorical data are presented as numbers and proportions and were compared using the chi-square test or Fisher’s exact test, as appropriate. Differences between means or medians were compared using the unpaired Student’s *t*-test or Mann–Whitney U-test, depending on the distribution. Survival curves were generated using the Kaplan–Meier estimator and compared using the log-rank test. A Cox proportional hazards model was generated to assess factors associated with survival, including pre- and intra-operative variables (univariate analysis). The results are displayed as hazard ratios (HRs) or adjusted hazard ratios with 95% confidence intervals. All tests were two-tailed, with significance set at a probability level of 0.05. All statistical analyses were performed using IBM SPSS version 26 (IBM Corp., Armonk, NY, USA).

## 3. Results

### 3.1. Baseline Cohort Characteristics

In our study of 76 patients (Table 1), the mean age of patients at diagnosis was 4.3 years (0.1–18.4) and at first operation 5.8 years (0.4–20.2), with 34.2% aged under 3 years. The mean interval between age at diagnosis and surgery was 1.2 years. A higher proportion of boys were included in our study (60.5% vs. 39.5% of girls). Prematurity and genetic disorders were present in the same proportion of patients (14.5%) but were not correlated. Twenty patients (26.4%) had pre-existing bundle branch block, four (5.3%) of whom had complete bundle branch block. Most patients (77.6%) had other cardiac pathologies associated with SAS. Among the 14 patients (18%) with associated aortic valve disease, aortic regurgitation was the dominant form in 11 patients. Three patients had multilevel LVOT involvement (one arch, one supravalvular, and one valvular).

Previously, 12 patients (15.8%) underwent heart catheterization and 31 (40.8%) surgery (Table 2).

Among the patients who had undergone previous cardiac surgery, only two underwent surgery in outside hospitals for a diagnosis of SAS. Other previous surgeries included coarctation/interrupted aortic arch repair (n = 14), ventricular septal defect closure (n = 13), atrioventricular septal defect repair (n = 6), mitral valve repair (n = 4), and aortic valve repair (n = 2).

### 3.2. Echocardiographic Characteristics

In terms of ultrasound characteristics [Table 3], the peak gradient of the left ventricular outflow obstruction was 63.2 mmHg 63.2 (15.0–150.0). The mean aorto-septal angle was 123.8 (110.0–134.0). Half of the cohort had preoperative AR, but the vast majority had grade 1. The aortic valve morphology was tricuspid in 60 patients (17.1%). The mean distance between the lesion and valve was 6.8 mm in systole and 9.0 mm in diastole.

### 3.3. Surgical Procedure

All patients were operated through a median sternotomy, with cardiopulmonary bypass, normothermia, or mild hypothermia (32 °C). In addition to subaortic membrane resection, septal myomectomy, starting at the nadir of the right coronary cusp and extending counterclockwise to the nadir of the left coronary cusp, was performed with or without other associated cardiac procedures in 56 (73.7%) patients.

The younger the patient underwent surgery, the fewer myomectomies were performed: 8/16 (50%) for those under two years old and 48/60 (80%) for those over two years old (Fisher’s exact *p* = 0.04).

The aortic and mitral valve leaflets were invaded by fibrous tissue in 13 (17.1%) and 22 (28.9%) patients, respectively.

Among the entire cohort, the most common associated lesions addressed at surgery were accessory mitral tissue resection (n = 15), ventricular septum defect closure (n = 13), aortic valve repair (n = 11), and mitral valve repair (n = 5).

The mean times for cardiac bypass and aortic clamping were 76 min and 46 min, respectively. Only two patients underwent a second run of cardio-pulmonary bypass during the same procedure. In both cases, a residual ventricular septum defect was closed (iatrogenic, with complete atrioventricular block in one, and multiple ventricular septum defects including an “accessory” undetected preoperatively in the other). Additional surgical procedures for associated congenital cardiac anomalies (ventricular septum defect closure, atrial septum defect closure, and foramen ovale closure) were performed in 41 (53.9%) patients (Table 4).

None of the patients in this cohort underwent a modified Konno procedure. Intraoperatively, the modified Konno procedure was always kept as a plan B (rescue) in the event that the intraoperative post-repair TEE and/or invasive residual gradient measurements were not satisfactory.

In the discrete and fibro-muscular pattern (56 patients), nine patients (16%) had a peak gradient ≥ 25 mmHg after repair, and in the tunnel-like lesions (20 patients), two patients (10%) had a peak gradient ≥ 25 mmHg after repair. One patient underwent a third cardiac procedure (neonatal Norwood with Norwood take-down for biventricular repair and VSD closure later in infancy) in whom both valvular and subvalvular lesions were present. The residual gradient was mostly related to the valve. The other patient had an invasive LVOT gradient of 10 mmHg at the end of the repair. He had dynamic LVOT obstruction on day 3 postoperatively (60 mmHg), but it decreased to 20 mmHg on day 13.

### 3.4. Post-Operative Outcome

One patient in the cohort died during hospitalization (in-hospital mortality, 1.3%). A 2-year-old boy with a diagnosis of Tetralogy of Fallot and two previous sternotomies underwent an uncomplicated membrane resection associated with septal myomectomy. Within hours after surgery, the patient developed irreversible brain edema leading to early demise.

In terms of complications requiring surgical re-intervention, six patients (7.9%) required reoperation during hospitalization: four pacemaker implantations (5.3%), one redo of hemostasis (1.3%), and one mitral valve repair for damaged mitral leaflet after SAS resection (1.3%) (Table 5).

Postoperative complications included seven pleural effusions (9.2%), six pericardial effusions (7.9%), four complete atrioventricular blocks (5.3%), four pneumopathies (5.3%), two post-pericardiotomy syndromes (2.6%), and one mediastinitis (1.3%). In terms of ECG changes, six post-operative complete bundle branch blocks (7.9%) were diagnosed with no clinical repercussions. None of the patients developed stroke or chylothorax or had a residual septal shunt.

Regarding AR [Table 6], five patients (6.6%) had aortic valve function deterioration postoperatively: four patients had no leak and ended up with a 1/4 insufficiency, and one patient had a 1/4 insufficiency preoperatively and ended up with a 2/4 insufficiency. The degree of AR did not change after surgery in 56 patients (73.7%). In 15 patients (19.7%), AR improved by at least 1 degree on a scale of 4.

The early follow-up LVOT peak gradient (mmHg) was 9.9 ± 13.6 (mean ± SD).

### 3.5. Predictive Factors for Reintervention in Recurrence of SAS

Ten patients (13.1%) required reoperation for the recurrence of SAS. On average, these patients were younger than those who did not require reoperation (mean age 3.5 vs. 6.1 years, *p* = 0.078).

Of note, of those 10 reinterventions, 4 patients (25%) had their initial procedure performed under the age of 2 years and 6 were aged 2 years and older at their initial procedure (*p* = 0.11). Although neither weight nor body surface area at surgery were borderline predictive factors (*p* = 0.06), height at initial surgery was predictive of reintervention (*p* = 0.01) (Table 7).

The distance between the subvalvular lesion and the valve, in systole and diastole, could be a predictive factor for recurrence if more data are available.

There were no significant differences in terms of recurrence of SAS when analyzing preoperative variables, such as the presence of other associated cardiac pathologies, aortic valve morphology, calculated LV z-scores, or preoperative peak left ventricular ejection obstruction gradient (73 ± 25 mmHg vs. 62 ± 29 mmHg, *p* = 0.23).

A univariate analysis of factors predictive of recurrence revealed that smaller preoperative height and body surface area were risk factors for reintervention (*p* = 0.02 and *p* = 0.04, respectively) (Table 8).

**Figure 2:**
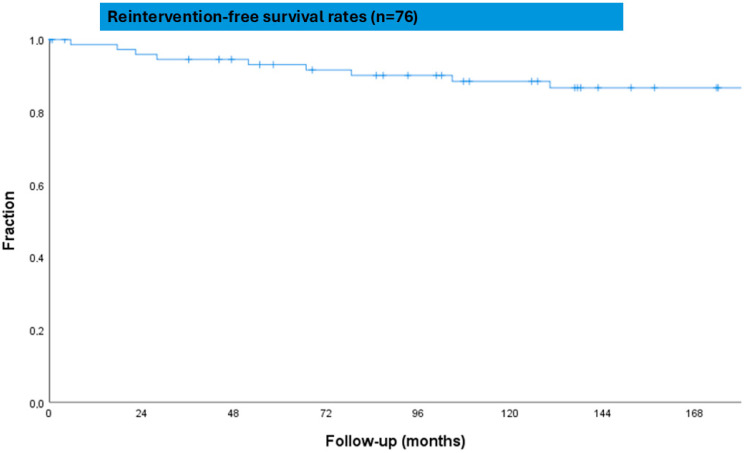
Kaplan–Meier survival curve for freedom from reoperation.

In our cohort, the reintervention-free survival rates at 5 and 10 years were 93.4% and 89.5%, respectively (Figure 2).

The reintervention-free survival rates at 5 and 10 years were 93.4% and 89.5%, respectively.

### 3.6. Survival

Three patients died during follow-up, at 70, 218, and 336 months, respectively. All causes were cardiac-related. Two of them had a recurrence of severe LVOTO and suffered cardiac arrest at home. The third patient died two weeks following a Ross procedure for severe aortic stenosis. She had Netherton syndrome (severe ichthyosis with chronic carriage of *multiresistant Staphylococcus)*. Her post-operative course was marked by Citrobacter septic shock and Pseudomonas aeruginosa mediastinitis, which led to death on postoperative day 18.

In our cohort, the overall survival rates at 5 and 10 years were 98% and 98%, respectively.

## 4. Discussion

Regarding our cohort population, 34.2% of our patients were younger than 3 years of age at the first surgery. Younger age at initial intervention has been associated with a higher reintervention rate in previous pediatric studies, hypothesizing that the phenotype of disease is more severe early in life [2,5,15,16,17]. We had a sex ratio in favor of boys, concordant with pre-existing literature [2,10,15,18]. Interestingly, we also observed a relatively high prevalence of genetic conditions [9].

Among our patients, 77.6% had associated cardiac lesions. This rate is comparable to the 80% reported in a recent study [5], but well above the 50–71% reported in earlier studies [9,10,18]. The repartition of our patients between the three subgroups of SAS was homogeneous and concordant with the existing literature, with discrete lesions being the most prevalent.

Regarding surgical aspects, myomectomy was performed in 74% vs. 16–57% in the literature [5,12], suggesting our surgical policy to be an aggressive approach. The preoperative peak gradients, mean 63.2 mmHg (15.0–150.0), were in line with adult recommendations for intervention. This gradient was higher in the subgroup requiring reintervention, but the difference was not statistically significant.

The surgical resection of SAS has a low operative risk, with an in-hospital mortality of 1.3% in our cohort, which is concordant with recent series [5].

The reoperation rate for complications was 7%, which was also consistent with the results of other studies [2,5,9]. Among the complications, conductive disorders requiring pacemakers were higher in our series than in others. We hypothesize that this is correlated with the high percentage of myomectomies (74%) associated with SAS resection. Nevertheless, only one case of “iatrogenic” ventricular septum defect was associated with this aggressive strategy.

Aortic regurgitation has been observed to progress over time in several previous publications [7,11,18,19], irrespective of surgical intervention. In our study, 5 patients (6.6%) had worsened pre-existing AR, but the majority of the patients had stabilized AR, and 15 patients (19.7%) showed improved AR after surgery. Damaging jets caused by the subaortic membrane or residual membrane tissue have been suggested as possible mechanisms for aortic regurgitation progression [2,9].

The recurrence rate for reintervention in our cohort was 13%, which is significantly lower than the 25–35% reported in other studies [5,9]. Our surgical strategy was aggressive in terms of the high rate of associated myomectomy in this cohort. We believe that one explanation for this low rate of recurrence is that a low postoperative gradient (mean < 10 mmHg) was achieved, as it has been shown that an increased postoperative pressure gradient constitutes a predictor of future reintervention [7].

Altered flow patterns due to a steepened aortoseptal angle may be a substrate for the development of SAS [20]. Although our mean aortoseptal angle was steeper (124°) than that in recent publications (136–138°) [19,21], we found no significant correlation with the risk of reintervention, probably because of the limited amount of data analyzed.

Our statistical analyses identified that the patient size at the time of surgery was the most significant predictive factor for reintervention. If we look into the percentages of SAS recurrence in function of the distance to the valve in diastole or systole, we noticed 3/8 (37.5%) and 3/10 (30%), respectively, for the “distal” lesions, against 4/20 (20%) and 2/13 (15.4%) for the lesions closer to the aortic valve (less than 8 mm). Even if there is no statistical difference due to the small cohort, recurrence is globally twice as frequent in lesions at 8 mm or more from the aortic valve. This may be clinically relevant.

However, this finding contrasts with other studies [10,12,15]. We hypothesized that a longer distance from the lesion to the aortic valve would make total surgical resection more difficult, with a risk of incomplete resection. Flow turbulence may persist at this level and increase the risk of recurrence.

Differentiating patients according to anatomical subtype is important to adopt correct clinical and surgical strategies in SAS treatment. Unfortunately, the definition of complex SAS, in contrast to the discrete membranous form of SAS, lacks uniformity in the revised literature. Therefore, the more aggressive surgical approach is difficult to generalize. Although the recurrence rate of SAS in this study was lower than that in some other series, several studies, including ours, have shown that septal myomectomy combined with resection of the subvalvular membrane is not a protective factor for reintervention [5,16]. Anatomical variations in the left ventricular outflow tract and an architecture that results in turbulent blood flow may constitute factors that are not completely correctable through surgery. Another theory suggests that scar tissue formation in the subvalvular area during the healing process leads to a fixed LVOT size, resulting in localized hypertrophy and fibrosis of the LVOT [22].

In this series, we did not perform any modified Konno procedure, which is indicated for diffuse SAS without significant aortic valve pathology, such as patients with severe forms of hypertrophic obstructive cardiomyopathy and children with tunnel SAS and a normal aortic orifice [23]. The modified Konno operation enlarges the conal septum with a prosthetic patch and is very efficient in relieving multilevel LVOT obstruction without aortic valve replacement. However, postoperative complete atrioventricular block is common, and late-term outcomes are poor in terms of reintervention rates [24,25].

This underlines the complexity of managing this pathology and the need for case-by-case assessment when deciding the type of intervention to be performed. Therefore, it is essential to carry out a thorough preoperative discussion to establish a surgical strategy tailored to the individual characteristics of patients to minimize both the risk of recurrence and the risk of iatrogenic complications.

Furthermore, the 5- and 10-year reoperation-free survival rates were high, at 93.4% and 89.5%, respectively, and comparable to larger cohorts [17].

In the future, it would be of great interest to determine detailed criteria for the optimal surgical method and timing of intervention, especially in infants.

The major limitation of our study is that it was a single-center, retrospective study of a small cohort. We acknowledge that, due to the retrospective nature of the echographic imaging data acquisition, we only had a minimal dataset of preoperative complete and accurate echographic measurements in this study, which makes more advanced statistics difficult. The results of the statistical tests should be considered exploratory. We only considered the recurrence requiring surgical intervention and not the recurrence of the disease followed conservatively. This group of patients without intervention is also particularly of interest in determining the predictors of less severe recurrence. Another limitation of this single-center review is that it reflects a surgical policy that might differ from other centers, thereby limiting the generalizability of the results.

Finally, with a median follow-up time of 5 ½ years, we could not explore the long-term results of SAS repair and cannot extrapolate from our results. Previously, others have shown indications for reintervention up to 17 years after initial surgery for SAS [22]. We restricted the study to those aged <18 years.

## 5. Conclusions

Surgical resection is an effective treatment for SAS, with excellent survival rates. We emphasize that associating myomectomy minimizes the postoperative pressure gradient but carries a higher risk of AV block. However, myomectomy alone did not prevent disease recurrence. We confirmed that a younger age at the first intervention was a predictor of reintervention. Individualized assessment for surgical decision-making is therefore mandatory.

## Figures and Tables

**Table 1:** Baseline cohort characteristics.

Patient Characteristics	n = 76
Age at diagnosis (years), mean (range)	4.3 (0.1–18.4)
Age at surgery (years, mean (range)	5.8 (0.4–20.2)
Less than 3 years (%)	34.2%
3 years and over (%)	65.8%
Diagnosis–procedure interval (years), mean (range)	1.2 (0.1–8.9)
Sex (H/F)	46 (60.5%)/30 (39.5%)
Prematurity	11 (14.5%)
Chromosomic anomaly	11 (14.5%)
Down syndrome	3 (3.9%)
22q11 deletion	2 (2.6%)
Noonan syndrome	1 (1.3%)
Netherton syndrome	1 (1.3%)
Silver Russel syndrome	1 (1.3%)
Opitz syndrome	1 (1.3%)
Weight (kg) at surgery, mean (range)	20.6 (4.4–83.2)
Height at surgery (cm), mean (range)	105.5 (56.5–181.0)
Body surface area at surgery, mean (range)	0.74 (0.2–2.0)
Preoperative hypertension	6 (7.9%)
Left ventricular hypertrophy on ECG	28 (36.8%)
Previous bundle branch block	20 (26.4%)
Incomplete	16 (21.1%)
Complete	4 (5.3%)
Pacemaker	2 (2.6%)

**Table 2:** Associated cardiac disease.

Disease	Number (%), n = 76
SAS + Other cardiac diagnosis (total)	59 (77.6)
Ventricular septum defect	17 (22.4)
Aortic valve disease (stenosis/regurgitation)	14 (18.4)
Atrio-ventricular septum defect	14 (18.4)
Coarctation of the aorta	10 (13.1)
Shone complex	7 (9.2)
Tetralogy of Fallot	3 (3.9)
Persistent ductus arteriosus	3 (3.9)
Interrupted aortic arch	2 (2.6)
Atrial septum defect	2 (2.6)
Right ventricular outflow tract obstruction	2 (2.6)
Transposition of the great arteries with pulmonary stenosis	2 (2.6)
Mitral valve dysplasia	2 (2.6)
Hypertrophic obstructive cardiomyopathy	1 (1.3)
Previous catheterization procedure, *n* (%)	12 (15.8)
Previous cardiac surgery, *n* (%)	31 (40.8)

**Table 3:** Echocardiographic data.

Echocardiographic Variables	Number (%), n = 76
Aortoseptal angle (degree), mean (range)	123.8 (110.0–134.0)
Preoperative peak LVOT gradient (mmHg), mean (range)	63.2 (15.0–150.0)
Preoperative mean LVOT gradient (mmHg), mean (range)	37.1 (12.0–90.0)
Type of SAS	
Discrete	32 (42.1%)
Fibromuscular	24 (31.6%)
Tunnel-like	20 (26.3%)
Native aortic valve morphology	
Bicuspid	15 (19.7%)
Tricuspid	60 (78.0%)
Preoperative aortic stenosis	13 (17.1%)
Preoperative aortic regurgitation	46 (50.2%)
Grade 1	33 (34.4%)
Grade 2	7 (9.2%)
Grade 3	6 (6.6%)
Preoperative aortic valve stenosis and regurgitation	7 (9.2%)
LVEDD z-score, mean (range)	−0.1 (−7.9–11.3)
LVPWd z-score, mean (range)	1.0 (−7.8–13.6)
IVSd z-score, mean (range)	1.8 (−2.6–10.5)
Ejection fraction (%), mean (range)	77.0 (61.1–94)
Less than 25%	1 (1.3%)
Less than 50%	1 (1.3%)
Fractional shortening (%), mean (range)	42.9 (31.4–66.0)
AV annulus z-score, mean (range)	−1.1 (−5.6–2.5)
Lesion-AV distance, systole (mm)	6.8 (2.9–15.4)
Lesion-AV distance, diastole (mm), mean (range)	9.0 (4.0–17.0)
Less than 8 mm	19 (25.0%)
8 mm or more	10 (13.2%)

LVOT: left ventricle outflow tract, SAS: subvalvular aortic stenosis, LVEDD: left ventricle end-diastolic diameter. LVPWd: left ventricle posterior wall in diastole, IVSd: interventricular septum in diastole, AV: aortic valve.

**Table 4:** Surgical features.

Characteristics	Number (%), n = 76
Type of surgery	
Membrane resection	8 (10.5)
Membrane resection + myomectomy	36 (47.4)
Membrane resection + additional cardiac procedure	12 (15.8)
Membrane resection + myomectomy + additional cardiac procedure	20 (26.3)
Myomectomy	
Yes	56 (73.7)
No	20 (26.3)
Valvular involvement	
No	38 (50.0)
Aortic valve	13 (17.1)
Mitral valve	22 (28.9)
Aortic and mitral	3 (3.9)
Bypass time (min), mean (range)	75.9 (30–239)
Aortic cross clamp time (min), mean (range)	46.4 (19–172)
Second round of aortic cross clamp time, n (%)	2 (2.6)
Associated procedure type	41 (53.9)
Ventricular septal defect closure	13 (17.1)
Atrial septal defect or Patent foramen ovale closure	7 (9.2)
Konno or modified Konno	0

**Table 5:** Postoperative outcomes.

Characteristics	Number (%), n = 76
Duration of ventilation (hours), mean	10.5
Intensive care unit stay (days), mean	2.0
Length of stay at the hospital (days), mean	8.5
Reoperation for complication	6 (7.9)
Pacemaker	4 (5.3)
Bleeding	1 (1.3)
Mitral valve lesion	1 (1.3)
Postoperative complications	35 (46.1)
Pleural effusion	7 (9.2)
Pericardiac effusion	6 (7.9)
Post-operative bundle branch block	6 (7.9)
Post-operative atrioventricular block	4 (5.3)
Pneumopathy/sepsis	4 (5.3)
Syndrome post-pericardiotomy	2 (2.6)
Mediastinitis	1 (1.3)
In-hospital mortality	1 (1.3)

**Table 6:** Evolution of aortic valve regurgitation.

Aortic Valve Regurgitation	Number (%), n = 76
Improved	15 (19.7)
Same	56 (73.7)
Worsened	5 (6.6)

**Table 7:** Analysis between reoperation vs. no reoperation patient with SAS recurrence—Patients and echographic characteristics.

	No Reoperation (n = 66)	Reoperation (n = 10)	*p*-Value
Age at diagnosis (years), mean ± SD	4.7 ± 4.2	2.3 ± 2.1	0.10
Age at first surgery (years), mean ± SD	6.1 ± 4.5	3.5 ± 2.9	0.07
Interval diagnosis-procedure (years), mean ± SD	1.3 ± 2.0	0.6 ± 0.6	0.27
Weight at surgery (kg)	21.8 ± 14.8	12.9 ± 6.6	0.06
Height at surgery (cm)	58.1 ± 15.4	103.9 ± 24.0	0.02
Body surface area (m^2^)	0.7 ± 0.3	0.3 ± 0.1	0.06
Aortoseptal angle (degree), mean ± SD	123.4 ± 5.7	126.0 ± 3.4	0.39
Preoperative LVOT peak gradient (mmHg), mean ± SD	61.6–29.3	73.3 −25.2	0.23
Preoperative LVOT mean gradient (mmHg), mean ± SD	36.7–18.5	40.7–18.9	0.65
Native aortic valve morphology			0.32
Bicuspid, n (%)	12 (16.0)	3 (4)	
Tricuspid, n (%)	53 (70.7)	7 (9.3)	
LVEDD z-score, mean ± SD	0.0 ± 3.2	−0.9 ± 1.3	0.58
LVPWd z-score, mean ± SD	1.0 ± 4.0	0.5 ± 1.1	0.84
IVSd z-score, mean ± SD	1.8 ± 3.0	1.9 ± 1.8	0.95
Ejection fraction (%), mean ± SD	77.0% ± 7.2	76.7% ± 8.5	0.93
Fractional shortening (%), mean ± SD	42.5% ± 11.2	45.4% ± 6.4	0.42
AV annulus z-score, mean ± SD	−0.5 ± 2.5	−1.2 ± 1.7	0.24
Lesion-AV distance, systole (mm), mean ± SD †	7.9 ± 3.0	12.6 ± 6	0.02
Less than 8 mm, n (%)	11 (84.6)	2 (15.4)	
8 mm or more, n (%)	7 (70.0)	3 (30.0)	
Lesion–AV distance, diastole (mm), mean ± SD ‡	6.6 ± 3.0	9.5 ± 5	0.05
Less than 8 mm, n (%)	16 (80.0)	4 (20.0)	
8 mm or more, (%)	5 (62.5)	3 (37.5)	

LVOT: left ventricle outflow tract, SAS: subvalvular aortic stenosis, LVEDD: left ventricle end-diastolic diameter, LVPWd: left ventricle posterior wall in diastole, IVSd: interventricular septum in diastole, AV: aortic valve, † 23 patients with valid echographic imaging, ‡ 28 patients with valid echographic imaging.

**Table 8:** Univariable analysis of predictors of reoperation.

Characteristics	HR	95% CI	*p*-Value
Age at diagnosis	0.8	0.6–1.0	0.09
Age at procedure less than 2 years	3.1	0.9–11.2	0.07
Interval diagnosis—procedure	0.6	0.3–1.4	0.27
Weight at surgery	0.31	0.05–2	0.07
Height at surgery	0.69	0.5–1.0	0.02
Body surface area	0.1	0.01–0.9	0.04
Aortoseptal angle	1.09	0.9–1.3	0.39
Preoperative SAS subtype–fibromuscular and tunnel-like	1.9	0.5–7.4	0.35
Other left side lesions	1.0	0.2–3.9	0.99
Preoperative LVOT peak gradient > 60 mmHg	1.9	0.6–6.7	0.30
Preoperative aortic annulus diameter z-score	1.2	0.7–2.0	0.50
Valvular involvement	1.7	0.5–5.9	0.43
Mitral tissue	0.5	0.1–2.4	0.39
Surgical technique—Myomectomy	0.7	0.1–3.4	0.67
Early peak gradient post operation	1.04	1.0–1.09	0.07
Early follow-up LVOT peak gradient > 20 mmHg	1.8	0.5–7.0	0.38

## Data Availability

The data presented in this study are available on request from the corresponding author due to restrictions imposed by the institutional ethical committee.
